# The Natural History of Teneurins: A Billion Years of Evolution in Three Key Steps

**DOI:** 10.3389/fnins.2019.00109

**Published:** 2019-03-15

**Authors:** Ron Wides

**Affiliations:** The Mina and Everard Goodman Faculty of Life Sciences, Bar-Ilan University, Ramat Gan, Israel

**Keywords:** Arthropod, vertebrate, urBilaterian, Latrophilin, EGF, Ecdysozoa, chordates, TRIP (Teneurin-related immense protein)

## Abstract

The entire evolutionary history of the animal gene family, Teneurin, can be summed up in three key steps, plus three salient footnotes. In a shared ancestor of all bilaterians, the first step began with gene fusions that created a protein with an amino-terminal intracellular domain bridged via a single transmembrane helix to extracellular EGF-like domains. This first step was completed with a further gene fusion: an additional carboxy-terminal stretch of about 2000 amino acids (aa) was adopted, as-a-whole, from bacteria. The 2000 aa structure in Teneurin was recently solved in three dimensions. The 2000 aa region appears in a number of bacteria, yet was co-opted solely into Teneurin, and into no other eukaryotic proteins. Outside of bilaterian animals, no Teneurins exist, with a “*Monosiga brevicollis* caveat” brought below, as ‘the third footnote.” Subsequent to the “urTeneurin’s” genesis-by-fusions, all bilaterians bore a single Teneurin gene, always encoding an extraordinarily conserved Type II transmembrane protein with invariant domain content and order. The second key step was a duplication that led to an exception to singleton Teneurin genomes. A pair of Teneurin paralogs, Ten-a and Ten-m, are found in representatives of all four Arthropod sub-phyla, in: insects, crustaceans, myriapods, and chelicerates. In contrast, in every other protostome species’ genome, including those of all non-Arthropod ecdysozoan phyla, only a single Teneurin gene occurs. The closest, sister, phylum of arthropods, the Onychophorans (velvet worms), bear a singleton Teneurin. Ten-a and Ten-m therefore arose from a duplication in an urArthropod only after Arthropods split from Onychophorans, but before the splits that led to the four Arthropod sub-phyla. The third key step was a quadruplication of Teneurins at the root of vertebrate radiation. Four Teneurin paralogs (Teneurins 1 through 4) arose first by a duplication of a single chordate gene likely leading to one 1/4–type gene, and one 2/3-type gene: the two copies found in extant jawless vertebrates. Relatively soon thereafter, a second duplication round yielded the -1, -2, -3, and -4 paralog types now found in all jawed vertebrates, from sharks to humans. It is possible to assert that these duplication events correlate well to the Ohno hypothesized 2R (two round) vertebrate whole genome duplication (WGD), as refined in more recent treatments. The quadruplication can therefore be placed at approximately 400 Myr ago. Echinoderms, hemichordates, cephalochordates, and urochordates have only a single copy of Teneurin in their genomes. These deuterostomes and non-vertebrate chordates provide the anchor showing that the quadruplication happened at the root of vertebrates. A first footnote must be brought concerning some of the ‘invertebrate’ relatives of vertebrates, among Deuterostomes. A family of genes that encode 7000 aa proteins was derived from, but is distinct from, the Teneurin family. This distinct family arose early in deuterostomes, yet persists today only in hemichordate and cephalochordate genomes. They are named here TRIPs (Teneurin-related immense proteins). As a second of three ‘footnotes’: a limited number of species exist with additional Teneurin gene copies. However, these further duplications of Teneurins occur for paralog types (a, m, or 1–4) only in specific lineages within Arthropods or Vertebrates. All examples are paralog duplications that evidently arose in association with lineage specific WGDs. The increased Teneurin paralog numbers correlate with WGDs known and published in bony fish, Xenopus, plus select Chelicerates lineages and Crustaceans. The third footnote, alluded to above, is that a Teneurin occurs in one unicellular species: *Monosiga brevicollis*. Teneurins are solely a metazoan, bilaterian-specific family, to the exclusion of the Kingdoms of prokaryotes, plants, fungi, and protists. The single exception occurs among the unicellular, opisthokont, closest relatives of metazoans, the choanoflagellates. There is a Teneurin in *Monosiga brevicollis*, one species of the two fully sequenced choanoflagellate species. In contrast, outside of triploblast-bilaterians, there are no Teneurins in any diploblast genomes, including even sponges – those metazoans closest to choanoflagellates. Perhaps the ‘birth’ of the original Teneurin occurred in a shared ancestor of *M. brevicollis* and metazoans, then was lost in *M. brevicollis*’ sister species, and was serially and repeatedly lost in all diploblast metazoans. Alternatively, and as favored above, it first arose in the ‘urBilaterian,’ then was subsequently acquired from some bilaterian via horizontal transfer by a single choanoflagellate clade. The functional partnership of Teneurins and Latrophilins was discovered in rodents through the LPH1-TENM2 interaction. Recent work extends this to further members of each family. Surveying when the interacting domains of Teneurins and Latrophilins co-exist within different organisms can give an indication of how widespread their functional cooperation might be, across bilaterians. Paralog number for the two families is relatively correlated among bilaterians, and paralog numbers underwent co-increase in the WGDs mentioned above. With co-increasing paralog numbers, the possible combinatorial pairs grow factorially. This should have a significant impact for increasing nervous system complexity. The 3 key events in the ‘natural history’ of the Teneurins and their Latrophilin partners coincide with the ascendance of particularly successful metazoan clades: bilaterians; arthropods; and vertebrates. Perhaps we can attribute some of this success to the unique Teneurin family, and to its partnership with Latrophilins.

## Introduction

Teneurin family genes made their world debut about a billion years (Byr) ago (as argued below), and made their scientific debut 25 years ago in *Drosophila melanogaster* (Baumgartner and Chiquet-Ehrismann, 1993). Many aspects of Teneurins have been reviewed (Tucker et al., 2012; Ziegler et al., 2012; Leamey and Sawatari, 2014; Mosca, 2015; Woelfle et al., 2016). The family’s evolution has been well covered, with a broader understanding emerging from each newly found homolog and each newly completed genome (Tucker et al., 2012).

This present overview of Teneurin evolution benefits from two new game-changing sources of information. First, recent prokaryotic, and eukaryotic (especially those of metazoans), whole genome sequences beneficially fill in many previous evolutionary gaps. Mining those ‘gaps’ sheds considerable light on key steps in Teneurin evolution. Second, two publications have recently solved the three dimensional structure of a large extracellular portion of Teneurins (Jackson et al., 2018; Li et al., 2018), and another recognized this as a domain block (Ferralli et al., 2018). We can now relate to the domains of these proteins with definitiveness never before possible. In comparing Teneurin homologs and near relatives, we have better focus and clarity for analyzing and dissecting Teneurin relationships.

As never before, this new information streamlines the story of the Teneurin family. I posit that the approximately one billion years of Teneurin history can be described as 3 essential steps: birth of urTeneurin in the urBilaterian; creation of the duplicates Ten-a and Ten-m in the urArthropod; and a quadruplication, in the earliest vertebrates, to create paralogs Tenm-1–Tenm-4. Otherwise, the shape and list of Teneurins is virtually invariant for 1 Byr.

Yet this history has three caveats that serve as tantalizing footnotes to the streamlined story. First, a distinct family of genes encoding 7,000 amino acid (aa) proteins ‘spun-off’ from Teneurins in early deuterostomes. Second, some further copies of paralogs have emerged, but only in select vertebrates and arthropods, evidently associated with whole genome duplications. Third, a non-bilaterian Teneurin exists in one of the two sequenced choanoflagellates.

## Materials and Methods

Annotated gene, transcript and protein records were collected for Teneurins. To be comprehensive, this was carried out for whole genome projects’ annotations. This was complemented with searches of NR to collect data from the directed cloning of genes, genome and transcriptome annotations, and more.

Whole assembled genomes were analyzed directly for evidence of Teneurin gene numbers, for validating annotations, and for detecting missed annotations or partial annotations. This mostly centered on probing genome assemblies with tblastn, searching with the longest protein sequences from closest relevant species. Most effective was the use of: Teneurin protein sequences with the EGF repeats excised; complete-Teneurin protein sequences; and TRIP protein sequences. Isolated EGF-like sequences and different specific Teneurin domains were used for specialized searches.

Unassembled genome traces (sequence reads that are unassembled, for any reason, or “sequence chaff”) were analyzed directly. This mostly centered on employing tblastn using protein sequences from closest relevant species. These searches were performed for evidence of Teneurin gene annotations missed, due to their absence from the genome assemblies. The effort in fact often focused on searches for portions of Teneurins that were missing in the assemblies, and therefore in the annotations.

Sequences were aligned with Clustal X, were compared by PAUP^∗^ (Phylogenetic analysis using parsimony), and were then displayed in an unrooted phylogram. In other cases, sequence alignments were done with Clustal Omega, and then were largely shown as rooted cladograms.

## Results and Discussion

### The Entire Evolutionary History of the Animal Gene Family Teneurin Can Be Summed Up in Essentially Three Key Steps: Sections I, II, and III

#### (I) The First Step: The First Teneurin Arose From a Unique Series of Fusions in a Shared Ancestor of All Bilaterians, Leading to Teneurin-Singleton-Genomes

Teneurin, the animal gene family, has members widely reported in triploblast-bilaterians (Tucker et al., 2012; Mosca, 2015), but never in diploblasts. Every sequenced bilaterian genome has at least one Teneurin, but no trace of them can be found in sequenced diploblast genomes to date. The only non-bilaterian Teneurin gene is found in one choanoflagellate, in the phylum considered to be the closest living phylum to that of the animal kingdom (Tucker et al., 2012). That exception is treated below as “footnote three” in section V.

**Figure 1:**
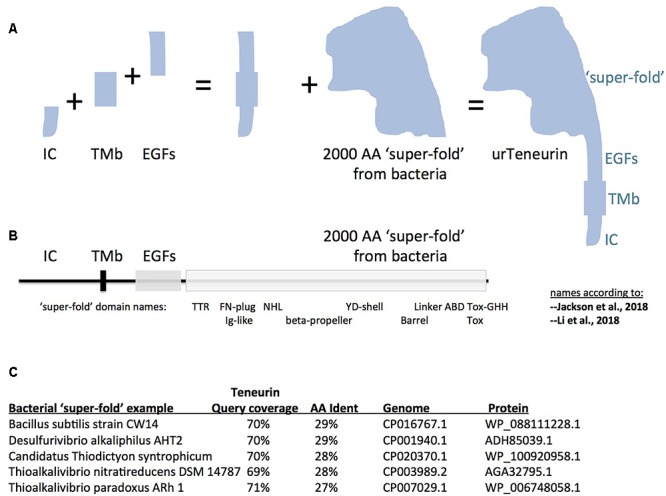
A model for “assembly-by-fusions” of the first Teneurin, or urTeneurin. **(A)** The emergence of Teneurin structure is modeled as a series of fusions of at least four genes. Protein domains encoded by these genes are diagrammed as four well recognized, discrete, Teneurin regions. “IC” is the intracellular domain, “TMb” is the transmembrane spanning region, and “EGFs” represents the seven or eight EGF-like repeats plus small associated domains. The “2000 amino acid ‘super-fold’ from bacteria” is a region recently recognized to share homology with dozens of proteins found in select prokaryotes. The ‘additions’ shown in the **(A)** diagram are not meant to imply a chronological order from left to right. **(B)** The four region names from panel A are reused above a linear representation of Teneurin. The extracellular region of Teneurin 2, as solved by Jackson et al. (2018) (chicken), and Li et al. (2018) (human), along with Teneurin 3 of mouse (Jackson et al., 2018), also had domain names assigned within the super-fold, as shown beneath the linear representation. TTR, transthyretin; FN, fibronectin; NHL, NCL/HT2A/Lin41 domain; YD, a repeat motif; ABD, antibiotic binding domain; and beta-propeller and barrel protein folds. **(C)** Five best homologies of Teneurin to prokaryotic proteins. Each have nearly 2000 aa acids of homology at more than a 27% amino acid identity level. The alignments start just after the Teneurin EGF-like repeats, and continue almost to the Teneurin carboxy terminus. In **(A)**, note that the shape of the cartoon “2000 aa ‘super-fold”’ is based on the extraordinarily highly concurring pictures of the structure from the Jackson paper and the Li paper, and therefore on the shape that evolution has delivered.

Teneurins encode type II transmembrane proteins composed of four distinct domain component regions: an intracellular domain; a single *trans*-membrane spanning domain; a extracellular domain with EGF-like repeats and its associates; and carboxy-terminally, a roughly 2000 aa ‘multi-domain entity’ whose three dimensional structure has been solved (Figure 1, Jackson et al., 2018; Li et al., 2018). Prior to these independent, and highly concurring, 3D solutions of this 2000 aa “super-fold” (Jackson et al., 2018), extracellular Teneurin domains were described using varied borders and domain names. The 2000 aa super-fold can be treated as one discrete unit in an evolutionary discussion, in that: it was adopted, all domains *en masse*, from a precursor gene encoding a bacterial protein; and it is essentially invariant among all Teneurins.

It is therefore most likely that the first Teneurin arose from an ancient small number of gene fusions that brought together the four mentioned component regions. A pair of fusions of a gene encoding a *trans*-membrane helix: to one encoding EGF-like repeats, C-terminally; and to one encoding an intracellular stretch, N-terminally; led to the type II membrane-orientation now found for every Teneurin (Figure 1A,B). The intracellular and transmembrane domains are highly variable and are poorly conserved between Teneurins. Therefore no homology can be found, nor relationships inferred, between these domains and non-Teneurin proteins’ domains.

In contrast, the affinities between Teneurin EGF-like repeats and analogous domains in other proteins and protein families *can* be examined. EGF-like repeats are nearly exclusively an animal phenomenon (Wouters et al., 2005). A number of EGF-like-domain bearing proteins have family orthologs that populate and ‘straddle’ both protostome and deuterostome genomes, so were likely present in the ‘urBilaterian.’ From among those ancient ‘cross-bilaterian’ proteins, the EGF-like repeats of Teneurin are most like those of Delta, Serrate/Jagged, Notch, Wif1/Shifted, Eyes-shut, Crumbs, Integrin beta2, and Slit. They are a subset of proteins that contain hEGFs, rather than cEGFs (Wouters et al., 2005). The gene fusion to include these EGF-like repeats into urTeneurin must have taken place in the urBilaterian, most likely by adopting EGF-repeats from one of these proteins. The protein bearing the most similar EGF-like block is most likely the one whose gene Teneurin “adopted from.” The eight EGF-like repeat content of Teneurin probably arose from a combination of: the integration of several adopted EGF-like encoding repeats as-a-block, plus accretion of repeats via tandem duplications (and possible repeat loss). Proposing candidate protein families as the most likely donors of Teneurin’s EGF-repeats will likely require modeling and testing a combination of these processes. Note: it is formally possible that Teneurins adopted EGFs from some no longer existing family, rather than one among the cluster above.

There is only one set of EGF-like repeats more similar to those of Teneurins, rather than those of the proteins listed above: the Tenascins. Tenascins arose in chordates, and are absent in any other phyla of animals, or life (Tucker and Chiquet-Ehrismann, 2009; Adams et al., 2015). Since Tenascins’ EGFs are closest to those of Teneurins, Tenascin’s EGFs most likely arose from deuterostome-chordate, Teneurin EGF-like domains. Genes encoding these EGFs must have fused with those encoding fibronectin type-III, and fibrinogen, domains, during Tenascin’s genesis. Historically, Teneurins therefore take their name from their ‘offspring.’ Another conceivable scenario is that Tenascins adopted EGFs from some no longer existing family, or from proteins extant only in lower chordates. There will be further discussion of this below, in section III.

Proceeding carboxy terminally, the remainder of Teneurin is the 2000 aa ‘super-fold.’ Since it is solved by cryo-EM and X-ray crystallography, the super-fold can now be coherently described using the nomenclature of these structure-solution papers. The terms used now are able to anchor the domains within the super-fold onto clearly-delineated 3D folds. However, the folds are named differently by the two groups: TTR-FN-plug/Ig-like; NHL/beta-propeller; YD-shell/Barrel, ABD, and Tox GHH [see Figure 1B, as superimposed basically on chicken/human Teneurin-2, (Jackson et al., 2018; Li et al., 2018)]. Subsequent to, or concomitant with, the gene fusions described above, this greater than 2000 aa structure was co-opted as-a-whole from bacteria, and was fused downstream of the EGF-like repeats. The wholesale adoption of this huge block of domains was not recognizable until recent bacterial genomes were sequenced. Some 100 newly sequenced genomes of bacterial species, collected mostly since 2016, encode proteins with this 2000 aa block. In the 100 cases, sequences of Teneurin (for instance of *M. brevicollis* or Ten-m of *D. melanogaster*) have about 70% of their length covered at more than 25% aa identity to the prokaryotic proteins. The homology starts just after the Teneurin EGF repeats, and covers nearly the entire post-EGF length of Teneurin. Five of the best hits are presented in Figure 1C. One of these hits (Figure 1C) was recognized as an analogous prokaryotic ‘super-fold’ containing protein in *Bacillus subtilis* strain CW14 in the structure paper (Jackson et al., 2018). The Teneurin extra-cellular regions’ structural homology to bacterial Tc-toxins is pivotal in the other structure paper (Li et al., 2018). A recognition that the homology between Teneurin and bacterial proteins extends to a full 2000 aa was also made in Ferralli et al. (2018). The *Desulfurivibrio alkaliphilus* YD protein discussed there also appears among the best hits shown here in Figure 1C. Note that before these recent abundant new bacterial genes were sequenced and recognized, Teneurins at best had RhS and YD-repeat portions with noticeable homology to bacteria. These previous alignments were shorter and localized (Minet and Chiquet-Ehrismann, 2000; Tucker et al., 2012). Those alignments did not suggest a wholesale adoption of the post-EGF portion of Teneurins as a block.

##### The overall outlook

A limited number of gene fusions led to the first Teneurin, in an ancestor of both Deuterostomes and Protostomes. Its original domain content and order strongly resist changes, as seen for all family members.

#### (II) The Second Step: A Single Gene Duplication at the Root of Arthropod Radiation Gave Rise to Paralogs Ten-a and Ten-m

**Figure 2:**
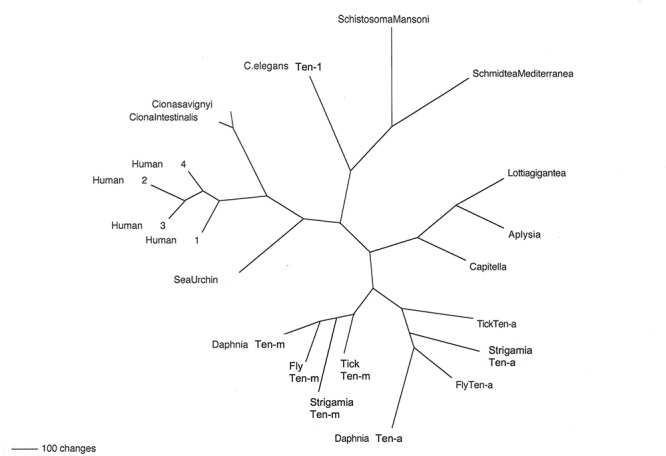
Teneurins from selected representatives of metazoans. An unrooted phylogram of Teneurin protein sequences shows the relatedness of examples from several phyla. Starting with Deuterostomes, on the left side of the figure, the Teneurins that appear are as follows: that of the Echinoderm *Strongylocentrotus purpuratus* (Sea Urchin); those of the Tunicates *Ciona intestinalis* and *Ciona savignyi*; and the four Teneurin paralogs of *Homo sapiens* (Human 1–Human 4). Continuing clockwise, Teneurins appear for the Nematode *Caenorhabditis elegans* (*C. elegans* Ten-1), for the flatworm Platyhelminthes *Schistosoma mansoni* and *Schmidtea mediterranea*, for the Molluscs *Lottia gigantea* and *Aplysia californica*, and for the roundworm Annelid *Capitella telata* (Capitella). The two paralogs Ten-a and Ten-m each appear in every Arthropod: for the tick *Ixodes scapularis* (Tick), the millipede *Strigamia maritima* (Strigamia), the fruit fly *Drosophila melanogaster* (Fly), and the water flea Crustacean *Daphnia pulex* (Daphnia).

**Figure 3:**
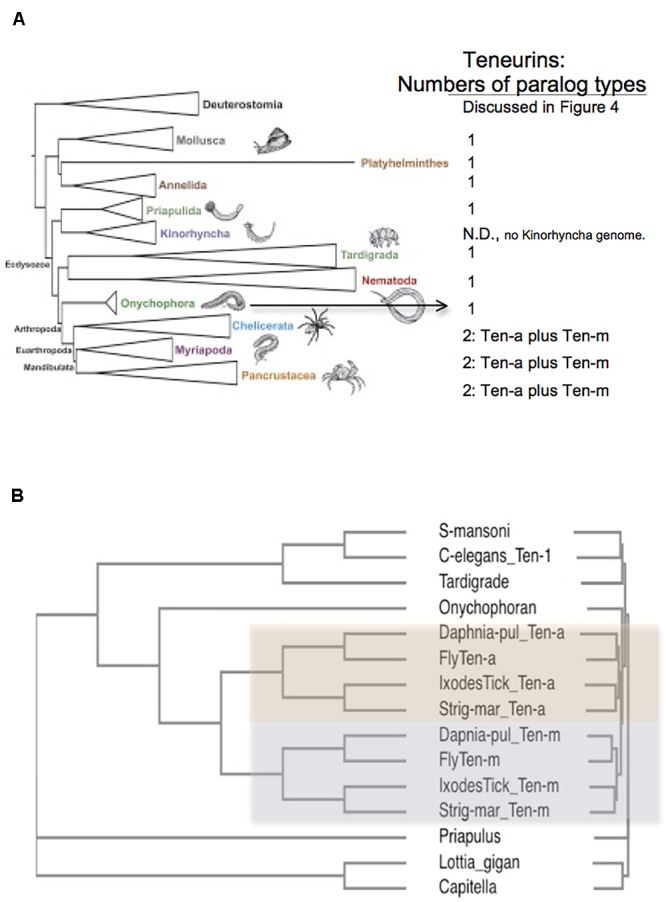
Arthropods have two Teneurin paralogs, Ten-a and Ten-m, while all other protostome genomes have one ‘singleton’ Teneurin. **(A)** A representation of Ecdysozoan phyla, as ordered in a graphical abstract of Borner et al. (2014) in *Molecular Phylogenetics and Evolution*. Deuterostomes, and some non-Ecdysozoan phyla, including Annelids, Platyhelminthes, and Molluscs, also appear. The insects and crustacean sub-phyla are represented together as “Pancrustacea.” On the right side of the panel, the numbers of Teneurin paralog types found for protostome species are listed. Except for Arthropods, all protostome genomes bear one ‘singleton’ Teneurin. **(B)** Protostome Teneurin protein sequences were compared using Clustal Omega. Teneurins from the following species were used: *Schistosoma mansoni* from Platyhelminthes, *Caenorhabditis elegans* from Nematodes, *Hypsibius dujardini* (a Tardigrade), *Euperipatoides rowelli* (an Onychophoran), *Priapulus caudatus, Lottia gigantea* from molluscs; and *Capitella telata* from Annelids. For Arthropods, Ten-a and Ten-m were used from *Ixodes scapularis* (Ixodes Tick), the millipede *Strigamia maritima* (Strig-mar), the fruit fly *Drosophila melanogaster* (Fly), and the water flea *Daphnia pulex* (Daphnia-pul). Arthropod Ten-a and Ten-m appear on a colored background. Note that even the ‘sister phylum’ of Arthropoda, Onychophora, has a singleton Teneurin.

The genomes of protostomes contain a single Teneurin gene (Tucker et al., 2012) (Figure 2). The marked exception occurs in a single clade, the Arthropods, in all of its species’ genomes. The genomes of species in the Arthropod Phylum contain two Teneurin paralogs, *Ten-a* and *Ten-m* (Figure 2, 3B). Representatives shown for each sub-phylum (Figure 2, 3) have a Ten-a, and a Ten-m, gene: the fruit fly *Drosophila melanogaster* for insects in the sub-phylum of Hexapoda; the tick *Ixodes scapularis* for the class of arachnids, in the sub-phylum Chelicerata; the water flea *Daphnia pulex* for the class of branchiopoda, in the sub-phylum Crustacea; and the millipede *Strigamia maritima* for the class of millipedes, in the sub-phylum Myriapoda. A single duplication of an ancestral protostome Teneurin gene before divergence of the four sub-phyla, yielding Ten-a and Ten-m, is consistent with the trees’ geometries. The relative phylogenetic distances between Ten-a and Ten-m are appropriate to the accepted evolutionary history of the four sub-phyla (Borner et al., 2014; Chipman et al., 2014; Misof et al., 2014).

All genomes of species from other protostome phyla contain a single Teneurin (Figure 2, 3). This statement includes all lophotrochozoa. More importantly for this discussion, it also includes all non-arthropod genomes examined from among the ecdysozoan cluster phlya of protostomes. Ecdysozoan genomes are generally widely represented, despite the fact that there are still some ecdysozoan phyla with no sequenced genomes: Nematophora (horsehair worms); Kinorhyncha (mud dragons); and Loricifera. A ‘Teneurin-singleton’ only is found in every sequenced species of non-arthropod ecdysozoans, in: Priapulida; Nematodes; Onychophorans, and Tardigrades (Figure 3A,B).

The most diagnostic information for timing the Ten-a/Ten-m duplication comes from the closest, sister, phylum of Arthropods, Onychophora. It has one species with a sequenced genome, *Euperipatoides rowelli* (NCBI BioProject 203089, Georg Mayer at the Baylor i5K initiative, as described in Evans et al., 2013). This Onychophoran velvet worm *E. rowelli* has a single Teneurin gene, roughly equally distant from fly *Ten-a* and from *Ten-m* (Figure 3). Onychophorans have an estimated divergence date from Arthropods not long before the Arthropod splits to sub-phyla (Sanders and Lee, 2010). The generation of a Teneurin paralog type occurred once in the history of invertebrates, exclusively in Arthropods, at their inception as a phylum.

##### To summarize

Genomes published to date firmly point to a single very ancient Teneurin duplication during all of protostome evolution, in the urArthropod. The four hugely populated extant sub-phyla of the arthropods then inherited the two paralogs from the proto-urArthropod ancestor. The duplication thus occurred in the proposed brief period between the Onychophoran/Arthropod split and the split of Arthropods to four sub-phyla (or perhaps to more sub-phyla, including trilobites) – in the early Cambrian, in the Ediacaran, or earlier yet. Previous reports that did not detect this deep origin of Ten-a and Ten-m were dependent on many fewer completed arthropod genomes (Tucker et al., 2012).

As described below in IV, the only other instances, whatsoever, of more Teneurin genes in protostomes occur in select Arthropods. These are additional Ten-a and Ten-a copies that arose much later, in limited lineages of chelicerata and crustaceans, as the result of evident whole genome duplications (WGDs). As documented below, appearance of these additional Ten-a’s and Ten-m’s correlate to known WGDs.

##### The overall outlook

Teneurins are unchanging over evolutionary time. An invariant protein architecture, together with only a single lasting duplication over 700 Myr of protostome evolution, attest to a conserved role and unchanging placement in an otherwise dynamic interactome/proteome landscape.

#### (III) The Third Step: Events Early in Chordate-Related Lineages, Plus Later Independent Vertebrate Duplications That Gave Rise to the Vertebrate Paralogs Teneurin 1, 2, 3, and 4

The third milestone in the history of Teneurins, after their appearance in the urBilaterian, and after their duplication in the urArthropod, occurred in deuterostomes around the establishment of the chordate phylum. Most central and relevant to our topic was a quadruplication in vertebrates that led to the four long-recognized Teneurin paralog types in higher vertebrates: 1, 2, 3, and 4. The invariant existence of these four paralog types in all higher vertebrates has already been well reviewed (Tucker et al., 2012; Leamey and Sawatari, 2014; Mosca, 2015).

**Figure 4:**
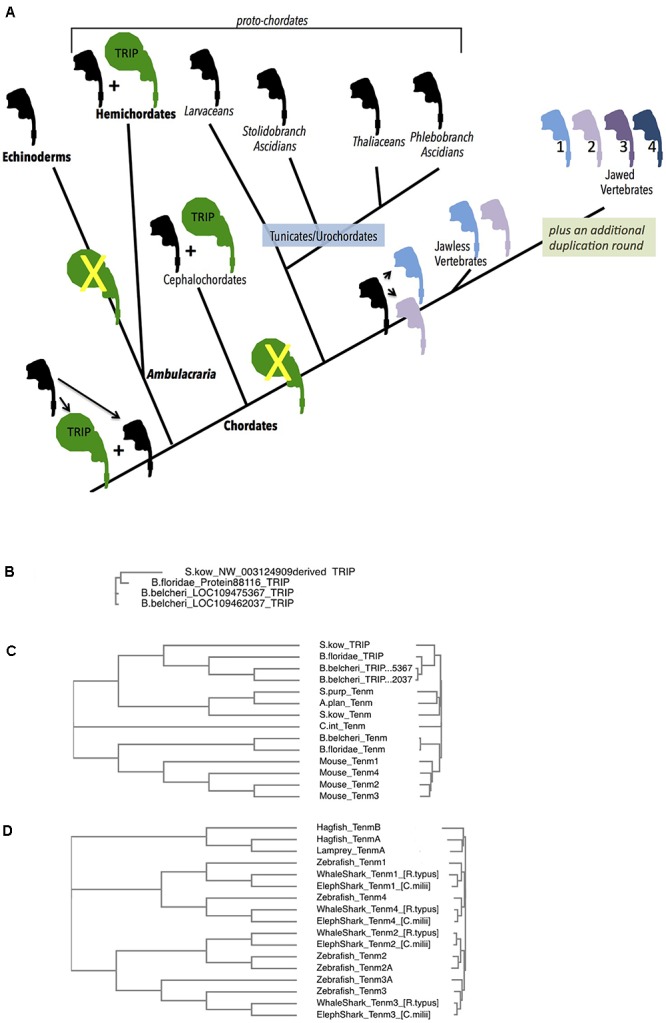
Deuterostome history of Teneurins, displaying the rise of the TRIP family in protochordates, and the quadruplication of Teneurins in vertebrates. **(A)** A diagram summarizing the Deuterostome history of Teneurins and TRIPs (Teneurin related immense proteins) use the Teneurin shaped icon “assembled” in Figure 1A. A black icon represents the Deuterostome ancestral Teneurin singleton and all singleton Teneurins on the tree. The larger TRIP icon is green, and represents the larger, 7000 aa TRIP protein. Teneurin paralogs in jawless and jawed vertebrates are products of duplications, and appear in color. Phylum names appear with bold letters, and sub-phylum and class names are not bold. Larger groupings (proto-chordates and Ambulacraria) also appear. **(B)** A Clustal Omega alignment of four TRIP proteins in their entirety. They each have a greater than 6500 aa length, and align from end to end with high homology. One TRIP is from the hemichordate acorn worm *Saccoglossus kowalevskii* (S. kow), one is from the cephalochordate lancelet *Branchiostoma floridae*, and two are from the lancelet *Branchiostoma belcheri*. The *S. kowalevskii* protein annotation had to be constructed ‘manually’ to overcome a large assembly inversion error in its host genomic scaffold. One of the two *B. belcheri* protein annotations had to be constructed ‘manually’ from adjacent contigs in the genome assembly. The three hemichordate TRIP proteins from the hemichordate acorn worm *Ptychodera flava* are not included in this alignment, but are fully homologous, and are much closer to the *S. kowalevskii* sequence. The cephalochordate *Asymmetron lucayanum* TRIP is not shown, but is more homologous to the other lancelets’ TRIPs. **(C)** Deuterostome Teneurins are aligned together with TRIP proteins. Only the N-terminal 2700 aa of the TRIPs are used for the alignment. TRIPs’ sequences that overlap Teneurins strongly partition away from Teneurins. The species used include those in **(B)**, plus the sea star *Acanthaster planci*, the sea urchin *Stronglyocentrotus purpulatus*, the tunicate *Ciona intestinalis*, and the four mouse Teneurins. **(D)** A Clustal Omega alignment of Teneurins from agnathans (jawless vertebrates), sharks, and fish. The species used were *Rhincodon typus* (elephant shark), *Rhincodon typus* (whale shark), *Danio rerio* (zebrafish), *Eptatretus burgeri* (agnathan, hagfish), and *Petromyzon marinus* (agnathan, lamprey). Two Teneurin homologs exist in the two agnathans, and were called “TenmA and TenmB.” The TenmB protein of lamprey was too incomplete to use effectively, so it was not included in the alignment. The two Teneurins aligned from hagfish, and the one aligned from lamprey are incomplete protein annotations.

The window to reconstruct earlier events for deuterostome Teneurins has now opened yet further, due to recently completed genomes. This includes views of proto-chordates: Ambulacraria (Echinoderms plus Hemichordates) and cephalochordates (Figure 4A). This also includes Tunicates (or urochordates), and ‘lower and higher’ – jawed and unjawed – vertebrates. As described below in IIIC of this section, the duplications of *bona fide* Teneurins only began in jawless vertebrates. However, a Teneurin-derived distinct family, the TRIPs, arose in deuterostomes before the appearance of vertebrates. There are extant TRIPs only among hemi- and cephalo- chordates. This TRIP sidetrack occurred first, and its narrative is treated first, in sections IIIA and IIIB.

##### (IIIA) The Echinoderm phylum and the Hemichordate phylum. A TRIP sidetrack

At their divergence from protostomes, deuterostomes also had an evident single Teneurin gene in their genomes. The several sequenced genomes of Echinoderms: including those of starfish, brittle stars, sea urchins and sea cucumbers, all have one Teneurin gene. This strongly supports a ‘singleton’ content for deuterostomes at their emergence.

In Hemichordates, the sister phylum of Echinodermata, a single Teneurin gene exits. This is the case for the two Hemichordate species with sequenced genomes, the Enteropneusta class acorn worms’: Harrimaniidae family’s *Saccoglossus kowalevskii*; and Ptychoderidae family’s *Ptychodera flava.* Each encodes a typical size Teneurin protein of approximately 2800 aa that is roughly equally homologous to arthropods’ paralogs Ten-a and Ten-m, or alternatively, to vertebrates’ paralogs Tenm-1, 2, 3, and 4 (Figure 4 and below). The remaining class of Hemichordates, Pterobranchians, have not been sequenced, so their Teneurin content is unknown.

However, there is an additional, novel, occurrence in both sequenced Hemichordate species that is striking. These Hemichordates contain genes encoding approximately 7000 aa polypeptides whose amino-terminal portions are unequivocally related to Teneurins (Figure 4). The genes in the two species are clearly homologous. I have named them Teneurin-related (or derived) immense proteins (TRIPs). Following approximately 2600 Teneurin-homologous amino acids, these polypeptides’ sequences continue carboxy-terminally with more than 4000 amino acids with no similarity to any eukaryotic proteins. Any similarity to bacterial proteins is in short scattered stretches, at very low homology. TRIPs evidently arose early in deuterostome/proto-chordate evolution from a duplicate of the ancestral singleton Teneurin, which then soon fused with a gene encoding very large protein coding domains. *S. kowalevskii* contains a single TRIP, and *Ptychodera flava* contains 3 TRIP paralogs that derive from further very ancient duplications. The *S. kowalevskii* protein shares more than 35% amino acid identity for over 6000 aa when compared to each of the three *P. flava* proteins.

Teneurin-related immense proteins’ polypeptides are among the largest ever described. They represent a large departure from Teneurins, qualifying them as a distinct protein family. The TRIP Teneurin-like-portions bear a lower level of homology to vertebrate and other deuterostome Teneurins than do the typical 2800 aa, *bona fide*, Teneurins of these Hemichordates (Figure 4C). This large TRIP/Teneurin distance shown in Figure 4C is based on the alignment of Teneurins with the first 2700 aa portions of TRIPs. Also rendering them distinct, the TRIPs lack amino acid stretches that are invariant according to the structures described in the publications establishing Teneurin structure (Li et al., 2018; Jackson et al., 2018). Conjectures about TRIP function appear below, in Section IIIB.

##### (IIIB) Non-vertebrate chordates: The TRIPs continue and end****

At the root of the chordate phylum, all genomes have a single *bona fide* Teneurin. Starting with the “proto-chordates,” the chordate sub-phylum Cephalochordata has three lancelet species with sequenced genomes: *Asymmetron lucayanum; Branchiostoma floridae;* and *Branchiostoma belcheri.* Each has a Teneurin (Figure 4A). At the same time, these cephalochordates bear TRIP genes (Figure 4A–C). Their phylogenetic relationships show that the cephalochordate TRIPs share their origins with the event that gave rise to Hemichordate TRIP genes, and are their orthologs (see Figure 4B, where the entire 7000 aa TRIP protein sequences are used for alignments). Two of these three lancelet species have a single TRIP. The third, *Branchiostoma belcheri*, has two TRIP proteins that share 98% amino acid identity with each other. This TRIP paralog pair is likely a duplication solely in *B. belcheri*, occurring after it diverged from *B. floridae* (Figure 4B, Yue et al., 2016).

Like cephalochordates, the chordate Tunicate sub-phylum has genomes bearing a single Teneurin gene (Figure 4). Unlike cephalochordates, however, urochordate/Tunicate genomes demonstrate no evidence of TRIP genes whatsoever. This is demonstrated in diverse examples distributed among three distant groupings within the Tunicate sub-phylum (Figure 4A). Of the Thaliacia class pelagic swimmers Salps, *Salpa thompsoni* has a single Teneurin and no TRIP. This genomic content is mirrored in an Appendicularia class pelagic swimmer Larvacean, *Oikopleura dioica*, in three Ascidian Phlebobranch sessile sea squirts *Ciona intestinalis*, *Ciona savignyi*, *Phallusia mammillata*, and a Stolidobranch ascidian *Botryllus schlosseri* (Voskoboynik et al., 2013). Unequivocally, TRIPs were lost on the path of the lineage to urochordates, after their divergence from more basal proto-chordates. As described below, this appears to be a momentous loss, as TRIPs also do not appear in any vertebrate (see Figure 4A–C, and below).

On TRIP structure: the first assumption is that TRIPs are type II proteins, but their sub-cellular deployment and function must be established and proven with further work. Some TRIP protein sequences have a transmembrane hydrophobic stretch in the position corresponding to that seen in Teneurins. Other TRIPs have no such transmembrane stretches. Missing transmembrane domains might be identified later when TRIP transcripts are isolated, once they are re-examined and better annotated. Likewise, TRIPs do not bear intracellular domains (IC) that correspond to those of Teneurins. Here too, though, better annotation of these genes, transcripts, and proteins might reveal that they contain IC stretches. Given the low sequence conservation of Teneurin ICs, this will take considerable work to investigate and establish. At the same time, however, some of the TRIP protein sequences do have a clear *trans*-membrane domain followed by EGF-like repeats, which begs the question if they can dimerize with *bona fide* Teneurins resident in the relevant species. It is also reasonable to ask if the additional extracellular domains, of more than 4000 amino acids, might render them more effective adhesion proteins? An additional question arises: might TRIPs ‘retain’ the ability to interact with LPHNs, or might there be some ‘replacement’ LPHN-like alternative for TRIPs.

On Tenascin evolution: it is possible that the EGF-like domains adopted by Tenascins have an alternative source – TRIPs, rather than Teneurins. Tenascins first appeared in lower chordates, so the source of their EGFs that fused with other relevant domains have two alternative closest “contemporaneous” sources: Teneurin EGFs or TRIP EGFs. An analysis of EGF-like domains of Tenascins, Teneurins, and TRIPs indicated that TRIP is the closer and more likely EGF-domain contributor (data not shown). Alignments and homology must be carried out more extensively, however, with care given to EGF-repeats as blocks. In the end, Teneurins might ultimately be named after their ‘grandchildren’ among their ‘offspring.’ The evolutionary sequence might well be: Teneurins gave rise to TRIPs, which then contributed EGFs to the ‘assembly’ of Tenascins. TRIPs were in the right time and phyla to have contributed EGF domains to the nascent family, Tenascins.

##### To summarize

The most parsimonious explanation in the early history of deuterostomes is that ‘the TRIP sidetrack’ began in the shared ancestor of Ambulacraria (Echinoderms plus Hemichordates) and Cephalochordates (Figure 4A). TRIPs rapidly became a distinct, non-Teneurin entity. The echinoderm branch evidently then specifically lost them. Subsequently, TRIPs persisted in cephalochordates, but were lost a second time in the entire Tunicate/Vertebrate lineage. Therefore TRIPs are exclusive to Hemicordates and Cephalochordates, (Figure 4A). Unlike Teneurins, they don’t appear to provide a function essential to general metazoan survival, since they are specific to select Deuterostomes, and proved dispensable on the journey to vertebrates. It is a challenge to consider what shared lifestyle needs constrained hemichordates and cephalochordates from losing TRIPs. Only when the character and expression of those TRIP c-terminal 4300 amino acids are studied can the unique role of TRIPs, as confined to these protochordates, be probed.

##### The overall outlook

Non-vertebrate chordates did not generate lasting duplicate *Teneurin* gene paralogs. Instead, a duplication soon followed by fusion to an enormous extracellular domain addition gave rise to a distinct family: TRIPs. The Teneurin-derived sequence of the proto-TRIP drifted significantly, so TRIPs are clearly identifiable as outliers to the homology range seen for Teneurins, and make no contribution to the history of Teneurin lineages.

##### (IIIC) Vertebrates and the quadruplication of Teneurin to yield the Tenm1 – Tenm4 paralogs

The earliest vertebrates – the agnathan, jawless, hagfish and lampreys - - each have genomes with two Teneurins, and no TRIPs. A single hagfish, *Eptatretus burgeri*, from the agnathan Myxiniformes order has a sequenced genome with two Teneurin genes (Figure 4A,D). The two sequenced lampreys, *Petromyzon marinus* and *Lethenteron camtschaticum*, from the agnathan Petromyzontiformes order, each also have two Teneurin genes (Figure 4A). All of these are equally distant to the *bona fide* Teneurin sequences in lower Deuterostomes, e.g., to the Tunicate *C. intestinalis*. This can correlate well with the model that these agnathans have undergone a single round of WGD, relative to basal chordates (Dehal and Boore, 2005; Caputo Barucchi et al., 2013; Berthelot et al., 2014). One step more modern in evolution, in jawed vertebrates, all four Teneurin paralog types exist, as has been extensively previously reported: Tenm-1 through Tenm-4 are seen in all species. Examples included here expand this foursome to Chondrichthyes (e.g., sharks and rays) and bony fish (Figure 4D). Thus, very early in the history of jawed vertebrates, a second duplication round yielded the -1, -2, -3, and -4 paralog types now found in all vertebrates. The two agnathan genes, named TenmA and TenmB for Figure 4, are present in every agnathan genome examined. However, the adequately complete protein annotations chosen for a meaningful multi-alignment were only *P*. *marinus* TenmA plus *E. burgeri* TenmA and TenmB (Figure 4D). The two agnathan paralogs A and B are roughly equally distant to the four higher vertebrate homologs. Agnathan TenmA and TenmB may partition to one 1/4-type and one 2/3 type, once better annotations and alignments are carried out. If so, the four Teneurin vertebrate paralogs (Teneurins 1 through 4) can be modeled as arising first by a duplication that led to the two genes seen in jawless hagfish and lampreys – one 1/4–type gene, and one 2/3-type gene, and thereafter by a second round duplicating each of these two. Whether or not it will be proven that one agnathan gene is a ‘more 2/3-type,’ and the other is a ‘more 1/4 type,’ the most parsimonious view is that these two led to the four higher vertebrate paralogs. In any case, a net quadruplication occurred within the short timeframe of the emergence of jawless, then jawed, vertebrates.

The Teneurin quadruplication that occurred at the root of vertebrates is integral to explaining the four extant vertebrate Teneurin paralog types. This net ‘double duplication’ happened long after the early TRIP ‘sidetrack’ that occurred in more ancient Deuterostomes that is described above. It can be argued that “true Teneurin” duplication events correlate well with the Ohno hypothesized 2R (2 round) vertebrate whole genome duplication (WGD) (Ohno, 1970). This hypothesis that early vertebrates underwent two rounds of WGD, followed by gene loss for a majority of duplicates, has been refined in many works (McLysaght et al., 2002; Dehal and Boore, 2005; Caputo Barucchi et al., 2013; Berthelot et al., 2014). Again, those works model agnathans as having undergone only the first of the two whole genome duplication rounds. The timing of the quadruplication is carefully modeled at approximately 400 Myr ago (McLysaght et al., 2002).

##### The overall outlook

The three steps of Teneurin family evolution harbored only three deep duplications in nearly a billion years of bilaterian evolution: one in the urArthropod; and two in very early vertebrates. The Byr time point is based on dates most ascribed to the divergence of protostomes and Deuterostomes (Nei et al., 2001; Blair and Hedges, 2005). This is a story of a protein and family profoundly resistant to change. Vertebrate duplications only occurred when the whole proteome complement duplicated. In fact, the only further Teneurin duplications observable in sequenced genomes are restricted to specific Vertebrate and Arthropod lineages, and appear to also be associated with Whole Genome Duplications (WGDs). They are isolated events, and are footnotes in comparison to the pivotal duplications described above as “steps 2 and 3” in Teneurin history.

### The Three Key Steps of Teneurin Evolution Are Accompanied by Three Important Footnotes. The ‘TRIP Sidetrack’ Footnote Is in Sections IIIA and B, Above. The Other Two Are Sections IV and V

#### (IV) Further Duplicates of Teneurins Are WGD Associated. Still an Unusually Unchanging Family

Beyond the two paralogs in arthropods and four paralogs in vertebrates, further Teneurin duplications are rare in the genomes sequenced to date, and only occur in a few very select Vertebrate and Arthropod lineages. The cases are limited to bony fish (teleosts) and amphibians, among vertebrates. None occur in cartilaginous fish, nor in the more modern vertebrates, Amniotes. Among Arthropods, they only occur in certain Chelicerata and Crustaceans. These duplications have arisen significantly more recently than the deep duplications described above, and always yield clearly identifiable paralog types 1, 2, 3, 4, a, or m.

**Figure 5:**
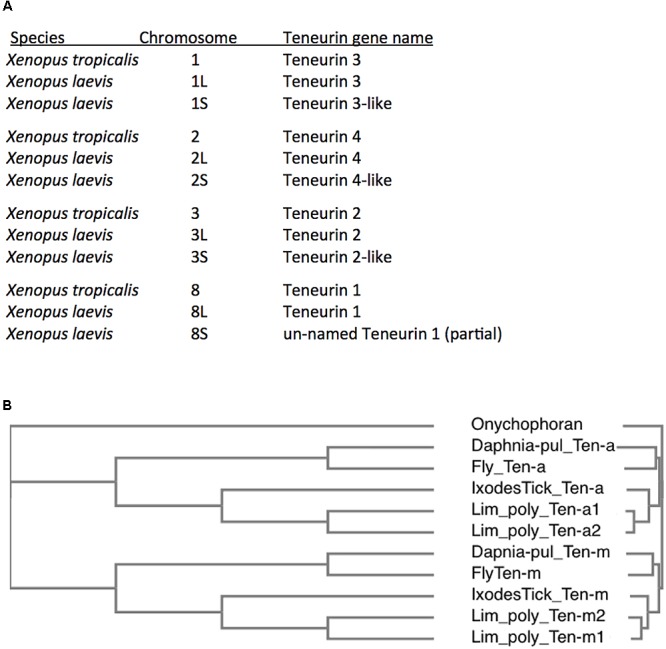
Additional Teneurin paralog copies have arisen in association with Whole Genome Duplications (WGDs) in specific vertebrates and arthropods. **(A)** A list of Teneurin genes in the diploid frog *Xenopus tropicalis* and in the allotetraploid *Xenopus laevis*. They are all shown with their chromosomal locations. *X. laevis* has duplicates of every Teneurin, and each one is located in syntenically conserved positions, relative to *X. tropicalis*. **(B)** A Clustal Omega alignment showing the duplication of Teneurins in the Chelicerate horseshoe crab *Limulus polyphemus*, that has two Ten-a and two Ten-m genes. It is compared to Onychophoran Teneurin, and to Teneurins of fly, tick, and water flea. As in Figure 3, the species used are *Ixodes scapularis* (Ixodes Tick), the fruit fly *Drosophila melanogaster* (Fly), and the water flea *Daphnia pulex* (Daphnia-pul).

The best known of these rare cases are in fish, modeled to have undergone teleost specific whole genome duplications (WGDs) (Pasquier et al., 2016). Well documented are the six Teneurins of the zebrafish *Danio rerio*, with a second Teneurin 2 paralog, and a second Teneurin 3 paralog (Howe et al., 2013). These duplicates share 81 and 74% aa identity, respectively, to their sister 2 and 3 genes, versus about 63% aa identity between paralogs Teneurin 2 and Teneurin 3. These extra paralog copies for Teneurins 2 and 3 are presumably those that persisted after a WGD, with loss of the Teneurin 1 and 4 duplicate-genes having occurred. Teneurin duplications are more extensive in Rainbow trout, whose genome is modeled as having undergone teleost specific, then salmonid lineage specific, WGDs (Berthelot et al., 2014). These two more recent lineage specific WGDs occurred long after the 2R genome quadruplication at the root of vertebrates. Rainbow trout have up to 16 Teneurins, all of identifiable types, Tenm1 – Tenm4. In amphibians, an even clearer case is seen when comparing diploid *Xenopus tropicalis* to the allotetraploid *Xenopus laevis* (Riadi et al., 2016). Like most vertebrates, *X. tropicalis* bears the four Teneurin vertebrate paralogs. *X. laevis* has clear second copies for each paralog (Figure 5A). This is especially compelling, as the eight *X. laevis* genes are nested in the expected syntenic surroundings, with pairs on the related chromosomes (allotetraploid pairings), as implicit in their chromosome names (Figure 5A).

Arthropods have the only other lineages showing duplications beyond those described in “Teneurin history steps two and three.” No further duplications are detectable in two of the four arthropod sub-phyla: neither in the one sequenced myriapod *Strigamia maritima*, nor in the hundreds of sequenced insects. Instead, additional duplications beyond the generation of the original urBilaterian Ten-a/Ten-m genes occur only in specific lineages of Chelicerates and Crustaceans.

In chelicerates, more than one paralog of Ten-m and Ten-a occur in specific mites, spiders, and horseshoe crabs. Both paralogs appear as multiples in all three sequenced scorpions and false scorpions. This is in contrast to the single Ten-a and Ten-m paralogs in the deer tick *Ixodes scapularis*, the Lyme disease vector known not to have undergone further WGDs. For the mite, spider, and horseshoe crab genomes with multiple Ten-m and Ten-a copies, the two paralog archetypes appear to always have the same duplication timing. This is based on the equivalent divergence tree profiles seen among the Ten-a and Ten-m duplicates, and likely indicates WGDs. An example is shown for the horseshoe crab *Limulus polyphemus*, that has two Ten-a and two Ten-m genes (Figure 5B). Compellingly, *Limulus polyphemus* and the other mentioned species have recently been fully sequenced, and are modeled as having had lineage specific WGDs (Nossa et al., 2014; Kenny et al., 2016; Schwager et al., 2017). The WGDs in horseshoe crabs, that occurred significantly after the original Ten-a/Ten-m split, are themselves still considerably ancient duplications (>135 Myr) (Nossa et al., 2014; Kenny et al., 2016). There is also a modeled deep specific WGD shared by spiders and scorpions (Schwager et al., 2017). These include the common house spider, *Parasteatoda tepidariorum*, with 6 Teneurins: three of each paralog (Schwager et al., 2017). A list of species examined is found online as Supplementary Table S1.

This story repeats itself in Crustaceans, with many distributed species showing more than one Ten-a and more than one Ten-m. It is notable that the class Branchiopoda of crustacea is one clade without any Teneurin duplications. In contrast, there are crustaceans with the two paralogs both duplicated or triplicated, with apparently the same timing. This strongly supports WGD. However, unlike for the case of Chelicerates, no crustacean genome sequenced to date has been modeled as having undergone WGD. This will require further investigation in the coming years to assess the full nature of these crustacean genomes and duplications.

##### To summarize

Duplications of Teneurins, after those that generated the long-recognized paralog types, appear restricted to cases of whole genome duplications. Those later duplications have occurred in specific Arthropod and Vertebrate lineages. They are the only metazoan phyla established in the literature to have undergone WGDs, outside of single-Teneurin lophotrochozoan Rotifers (Snell et al., 2015). After the Ten-a/Ten-m duplication, it appears that without exception, new Teneurin paralog generation is driven by whole genome duplication. The Ten-a/Ten-m duplication itself is so ancient in arthropod evolution that no known WGD can be invoked with which it could be associated.

##### The overall outlook

There appears to be little tolerance to generate and utilize new paralogs of Teneruins. It was already clear that Teneurin’s structure is unchanging. Now, as an extension: Teneurins also only change their ‘count’ when all other components of the proteome/connectome are duplicated, in WGDs. It is as if additional Teneurins are not suffered unless the entire proteome framework is preserved, via copying the entire endeavor. As such, Teneurin content is rigid relative to the proteome or connectome. It suggests that Teneurins themselves increase only when all their potential interactors increase too.

#### (V) Teneurins Are a Bilaterian-Only Metazoan Family, Except for One Choanoflagellate Clade

Teneurins are a bilaterian-only metazoan gene family, to the exclusion of the Kingdoms of prokaryotes, plants, fungi, and protists. The intriguing only exception comes from among the unicellular, opisthokont, closest relatives of metazoans, the choanoflagellates. Full genome sequences exist for only two choanoflagellates (Hoffmeyer and Burkhardt, 2016). There is a Teneurin in *Monosiga brevicollis*, as has been reported (Tucker et al., 2012). In contrast, the genome of the closely related *Salpingoeca rosetta* has no Teneurin.

To put these two contrasting choanoflagellate findings in context, it must be recognized that outside of triploblast-bilaterians, no animal Teneurins exist. There are no Teneurins in any diploblast genomes, including even sponges - those metazoans closest to choanoflagellates. This makes *M. brevicollis’* gene strikingly unique, and all the more intriguing. Perhaps its existence bespeaks horizontal transfer. One of the two most parsimonious explanations for *M. brevicollis’* gene’s existence is: the original Teneurin was ‘born’ in the shared ancestor of *M. brevicollis* and metazoans, then was lost in every diploblast metazoan lineage. This is possible, but is not an entirely satisfying explanation, due to the need for serial and multiple losses to “clear-the-board” in every branch. Alternatively, and favored above, is a model where urTeneruin was ‘fusion-assembled’ in the ‘urBilaterian,’ then was subsequently acquired by a single choanoflagellate clade, via horizontal transfer. Recently, nineteen additional Choanoflagellates had their transcriptomes extensively sequenced and compared (Richter et al., 2018). Evidence of Teneurins occur in 3 of the 19 species, essentially all clustered in one clade. As a comparison, signatures for domains of Notch and its ligands are found in the 19 transcriptomes, as well as in the two genome-sequenced choanoflagellate species. The conclusion reached is that Notch appears to be ‘indigenous’ to choanoflagellates, with its creation predating the choanoflagellate/metazoan split (Richter et al., 2018). In contrast, Teneurin distribution supports the idea that a metazoan Teneurin entered *M. brevicollis* and its clade with three other species, by horizontal transfer.

##### The overall outlook

The evidence is strongly biased toward an urBilaterian origin of Teneurin, followed by horizontal transfer into *Monosiga brevicollis*’ choanoflagellate clade.

### Teneurins and Latrophilins- Co-prevalence and the Importance of Their Co-existence: VI and VII

#### (VI) LPHN1-TENM2 Are the First Among Functional Partners. Expanded Families Offer Expanded Combinatorials for Interactions

The functional partnership of Teneurins and Latrophilins was discovered in rodents through the LPHN1-TENM2 interaction (Silva et al., 2011). Further work extends this to further family members, with demonstrations that all 3 latrophilins bind all 4 Teneurins, in Mouse (Boucard et al., 2014). A survey of where the interacting domains of Teneurins and Latrophilins co-exist within different organisms can give an indication of how widespread their functional cooperation might be across bilaterians. From protostomes to deuterostomes, how many of each exist?

First, what defines the Latrophilins, and where do they occur? Latrophilins exist as an animal-only gene subfamily within the greater seven-transmembrane GPCR family. They are part of the Adhesion GPCRs, of which there are 33 in humans. Adhesion GPCRs are one of five main groups according to the GRAFS classification (see Hamann et al., 2015). The Adhesion GPCRs are ancient, and are believed to have evolved from the cAMP receptor family, arising approximately 1,275 million years ago, before the split of Unikonts to animals and fungi (Schioth et al., 2010; Nordstrom et al., 2011; Krishnan et al., 2012). Among the adhesion-GPCRs, Latrophilins constitute family I out of 9 (I-IX) (Hamann et al., 2015). Family I LPHNs 1 through 3 are formally known as ADGR1-3, with the less related ELTD1 known as ADGRL4. There are no HRM, Olfaction, or RB-Lectin domains in ELTD1/ADGRL4, making it a distinct outlier. LPHN1 was the first Latrophilin cloned (Lelianova et al., 1997). Again, the interaction with Teneurin 2 was discovered as: LPHN1 to Teneurin2 in 2011, as mentioned above (Silva et al., 2011).

LPHNs are animal only genes that are distributed more broadly than Teneurins. In diploblasts, there is a strong possibility that homologs exist that truly act as LPHNs (Krishnan et al., 2014). Examples of these homologs include sponge *Amphimedon queenslandica* Aq715659, and anemone *Nematostella vectensis* Nv24490. Whatever the function of these proteins, it must be Teneurin-independent (Table 1).

**Table 1:** Teneurin and Latrophilin paralog numbers in animal genomes.

	Teneurins		Latrophilins
Diploblasts		0	1
Non-Arthropod Protostomes		1	1
*C. elegans*		1	1, or 2
Most Arthropods		2	1
Echinoderms		1	1
Hemichordates		1 (+TRIPs)	1
Cephalochordates		1 (+TRIPs)	1
Urochordates		1	0 or 1
Agnathan Lamprey		2	1
Most Jawed Vertebrates		4	3
*X. tropicalis*		4	3
Vertebrates and Arthropods that have undergone WGD:			
*X. laevis*		8	5
Zebrafish *D. rerio*		6	6
Rainbow trout		16	7
*Chelicerates with duplications*		4–6	2–10

Protostomes all seem to have one Latrophilin only (Table 1). Exceptions are a duplicate in the *Ixodes* tick, and a possible two for *Caenorhabditis elegans*, Lat-2, and perhaps also Lat-1, For insects, a singleton clear Latrophilin exists. Therefore a LPHN duplication does not appear to co-occur with the Ten-a plus Ten-m generated paralogs in insects. Some insect LPHNs seem to have a more complete domain content than others. Nonetheless, the *Drosophila melanogaster* homolog *Cirl*, evidently missing many expected domains, has clearcut functions in flies (Scholz et al., 2015; Scholz et al., 2017).

In non-vertebrate Deuterostomes, there are to one to two LPHNs, although generally only one has all domains to make a firm case for likely function (Krishnan et al., 2013). However, from chordates to vertebrates, a co-increase between Teneurins and Latrophilins can be tracked (Table 1). Most intriguingly, the clades of vertebrates and arthropods that underwent WGD and duplication of Teneurin paralogs have a definite trend of equivalent increases for the LPHNs. This can be seen for *Xenopus laevis*, Zebrafish, Rainbow trout, and Chelicerates (Table 1).

The LPHN genes’ duplications at the root of vertebrates/chordates could have co-arisen with the vertebrate whole genome 2R quadruplication proposed by Ohno (see Krishnan et al., 2015). WGDs in restricted arthropods and vertebrates appear to co-copy, and co-retain, their balanced collections of Teneurins and Latrophilins.

##### The overall outlook

The genome appears to duplicate Teneurins together with LPHNs. A balance of Teneurins to LPHNs, and to the overall content of the proteome and connectome, might need to be maintained. This suggests that the Teneurin content ratio is rigid, relative to gene counts in the genome, and especially to LPHN gene counts.

#### (VII) The Success of Vertebrates and Arthropods, and the Possible Contribution of These Two Families to That Success

There are three key steps to the Teneurin evolution story, the birth-by-fusions, then the very ancient duplication events. Three important footnotes serve either to bolster the character of those steps (e.g., conserved, specific additional duplications), or are not central to the generation of Teneurins as we know them (e.g., the TRIP spinoff, and a choanoflagellate’s homolog).

Teneurins are rigidly and extraordinarily conserved, both in their unchanging structure, and in their gene copy number per genome. The lineages where new paralogs arose are in the two most successful phyla on the planet: arthropods, and our own – chordate vertebrates. Is it possible that the addition of Teneurin paralogs to the gene toolkit contributed to the special success of these phyla? Can the overall success of triploblasts-bilaterians, compared to diploblasts, partially be attributed to the presence of Teneurin? Does a relatively fixed ratio of Teneurin to Latrophilin gene copies, conserved even when further arthropod and vertebrate WGDs occur, attest to the special advantage that this ‘team’ jointly contributes to a metazoan? These are all questions that could be further probed in the future, now that these balances have been uncovered.

The basic outline of this story should now be complete, and is even arguably comprehensive. Given the major gaps now filled in for the tree of animal life, large brushstrokes expected to change this story in the future seem unlikely. Albeit, some chapters of this story need improvement. A better-defined indication of the source of EGF-like repeats that were incorporated into Teneurin in the urBilaterian would be informative. An answer on whether Teneurins versus TRIPs provided Tenascins with their EGF-like domains should be more clear-cut with better modeling. Establishing a clearer relationship between agnathans’ two Teneurins and higher vertebrates’ four Teneurins is attainable with further work. Genome sequences of additional Choanoflagellates, and other unicellular opisthokonts, should shed more light on the significance of the *Monosiga brevicollis* Teneurin. The question: “just how rigidly conserved is the Teneurin family, relative to other gene families?”, should be interesting to model and probe. However, for all of these questions, it can only be expected that new genomes, and further connections within the omniome, will deliver new surprises.

## Author Contributions

The author confirms being the sole contributor of this work and has approved it for publication.

## Conflict of Interest Statement

The author declares that the research was conducted in the absence of any commercial or financial relationships that could be construed as a potential conflict of interest.
